# Supplementary material for: Hospitalisations Related to the Combination of ACE Inhibitors and/or Angiotensin Receptor Blockers with Diuretics and NSAIDs: A Post Hoc Analysis on the Risks Associated with Triple Whammy
Source: Healthcare (Basel). 2023 Jan 12;11(2):238. doi: 10.3390/healthcare11020238 (PMC9858958; doi:10.3390/healthcare11020238)

Supplementary Figure

Figure S1. Reporting odds ratios (RORs) of hospitalisation for all patients on triple whammy, according to age, sex, presence of a renal event, and class of suspected and concomitant medications

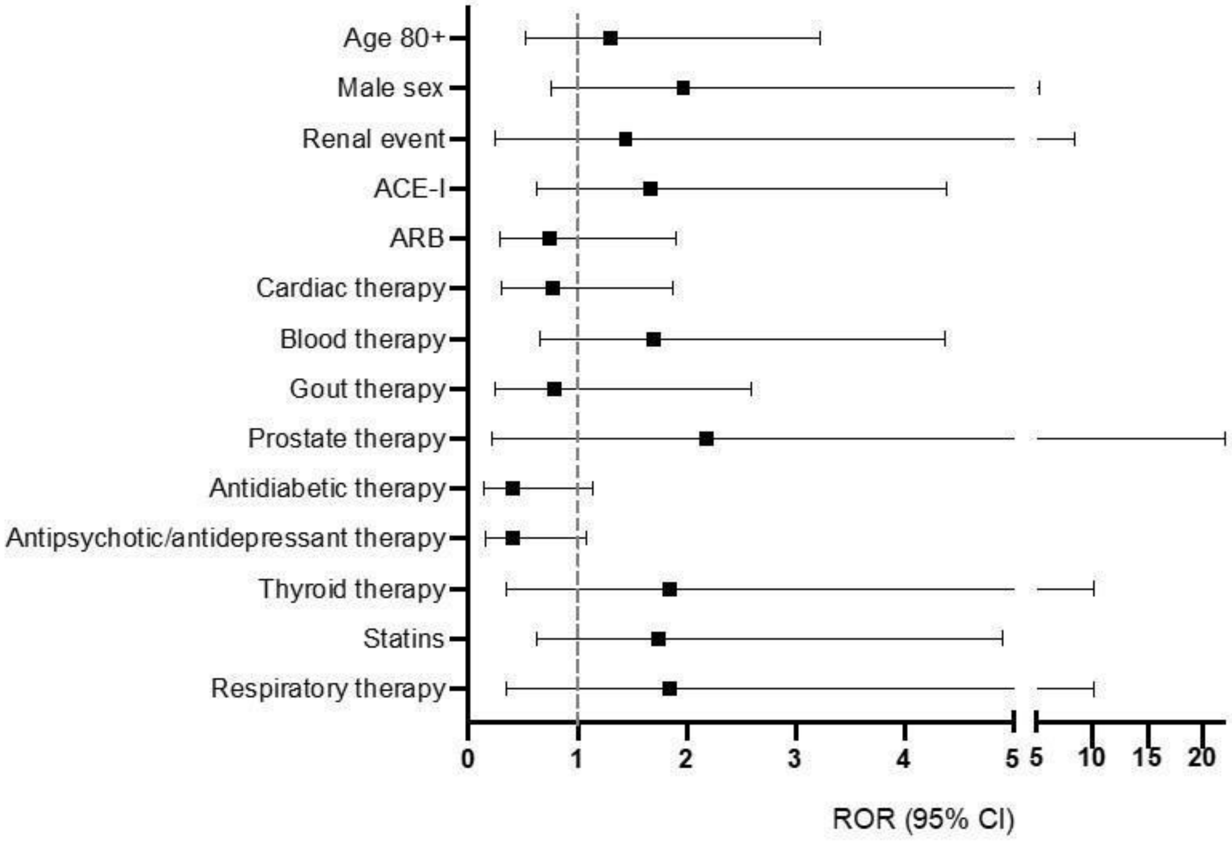

Supplement: Supplementary file 1 [file healthcare-11-00238-s001.zip › healthcare-2144568-supplementary.pdf]
